# Association Between Caregiver Strain and Self-Care Among Caregivers With Diabetes

**DOI:** 10.1001/jamanetworkopen.2020.36676

**Published:** 2021-02-11

**Authors:** Alexandra King, Joanna Bryan Ringel, Monika M. Safford, Catherine Riffin, Ronald Adelman, David L. Roth, Madeline R. Sterling

**Affiliations:** 1 New York Presbyterian Hospital/Weill Cornell Medicine, New York; 2 Division of General Internal Medicine, Department of Medicine, Weill Cornell Medicine, New York, New York; 3 Division of Geriatrics and Palliative Medicine, Weill Cornell Medicine, New York, New York; 4 Division of Geriatric Medicine and Gerontology, Johns Hopkins University School of Medicine, Baltimore, Maryland

## Abstract

**Importance:**

Caregiver strain has been shown to be associated with adverse effects on caregivers’ health, particularly among those with cardiovascular disease. Less is known about the association of caregiver strain with health behaviors among caregivers with diabetes, a disease that requires a high degree of self-care.

**Objective:**

To examine the association between caregiver strain and diabetes self-care among caregivers with diabetes.

**Design, Setting, and Participants:**

This cohort study was conducted between July 13, 2018, and June 25, 2020, using data on 795 US caregivers aged 45 years or older with self-reported diabetes from the Reasons for Geographic and Racial Differences in Stroke (REGARDS) study, which comprised 30 239 Black and White adults 45 years or older throughout the US enrolled from January 2003 to October 2007.

**Exposures:**

Caregiver strain, assessed by self-report in response to the question, *“*How much of a mental or emotional strain is it to provide this care?*”* Response options were no strain, some strain, or a lot of (high) strain.

**Main Outcomes and Measures:**

Diabetes self-care, which was assessed across 4 domains (Mediterranean diet adherence, physical activity, smoking status, and medication adherence), and a composite self-care score summing performance across these domains. The association between caregiver strain and diabetes self-care was examined with multivariable Poisson regression adjusting for demographic, clinical, physical and mental functioning, and caregiving covariates.

**Results:**

Among the 795 caregivers with diabetes included in the study, the mean (SD) age was 63.7 (8.6) years, 469 (59.0%) were women, and 452 (56.9%) were Black individuals. Overall, 146 caregivers (18.4%) reported high caregiver strain. In unadjusted models, high caregiver strain was associated with less physical activity (prevalence ratio [PR], 0.66; 95% CI, 0.45-0.97), low medication adherence (PR, 0.80; 95% CI, 0.68-0.94), and worse self-care (PR, 0.65; 95% CI, 0.44-0.98). In adjusted models, the association between some and high caregiving strain with low medication adherence remained significant (adjusted PR: some strain, 0.88 [95% CI, 0.78-0.99]; high strain, 0.83 [95% CI, 0.69-0.99]).

**Conclusions and Relevance:**

In this cohort study of US adult caregivers with diabetes, a high level of strain was associated with low medication adherence. Increased awareness of the prevalence of caregiver strain and potential ramifications on caregivers’ self-care appears to be warranted among health care professionals and caregivers.

## Key Points

**Question:**

Is there an association between caregiver strain and self-care behaviors among US caregivers with diabetes?

**Findings:**

In this cohort study of 795 caregivers with diabetes, those who experienced some strain or a high level of strain had significantly decreased medication adherence compared with those who experienced less strain.

**Meaning:**

The findings suggest that a high level of caregiver strain may be associated with decreased self-care behaviors among caregivers with diabetes.

## Teaser

This cohort study examines the association between the level of caregiver strain and self-care behaviors among US caregivers with diabetes.

## Introduction

At least 17.7 million adults in the US provide unpaid support to adults who are middle-aged or older.^1^ A report by the American Association of Retired Persons suggests that most of these unpaid caregivers are middle-aged women and 38% are members of minority groups.^2,3^ Often, they care for a spouse or other family member and assist with activities of daily living, instrumental activities of daily living, and sometimes medically oriented tasks. In fact, data suggest that unpaid family caregivers provide as much as 90% of the in-home long-term care needed by adults in the US.^4^ In the context of the coronavirus disease 2019 (COVID-19) pandemic, caregivers have become even more important, providing essential care to individuals with comorbid conditions and disabilities in the home.^5,6^

The demands of caregiving can leave caregivers vulnerable to mental and physical health problems.^4,7,8,9,10,11,12,13,14,15,16,17^ As such, the term *caregiver strain* (also known as caregiver burden) is defined as the extent to which caregivers perceive that caregiving has had an adverse effect on their emotional, social, financial, physical, and spiritual functioning.^18^ Studies have demonstrated that a high level of caregiver strain is associated with an increased risk of all-cause mortality and lower levels of perceived health compared with a lesser level of strain.^8,17,19,20^ In addition, caregiver strain is associated with poor cardiovascular health.^8^ Moreover, some researchers have shown that strained caregivers have higher Framingham Coronary Heart Disease and Stroke Risk scores and a higher incidence of metabolic syndrome compared with noncaregiving sex-matched control participants without coronary heart disease.^15,21,22,23,24^ However, these findings have not been wholly consistent. Other studies^25,26^ have revealed that a healthy caregiver phenomenon exists, whereby caregiving is associated with decreased mortality as compared with noncaregiving, perhaps suggesting that healthier persons are more likely to take on the caregiving role or that caregiving is associated with healthier behaviors.

Less is known about the association of caregiver strain with self-care practices for maintaining health by adult caregivers with diabetes. Recent reports suggest that nearly 20% of unpaid caregivers in the US have diabetes,^22,27,28^ a complex chronic disease that requires a high degree of self-care. Adults with diabetes are asked to maintain a healthy diet, exercise regularly, avoid smoking cigarettes, and take multiple medications for control of blood pressure and glucose and cholesterol levels.^29,30,31,32,33,34^ A systematic review^32^ of 72 studies of self-management among patients with diabetes revealed a positive association of self-management training with dietary habits and glycemic control. However, adhering to these recommendations and performing adequate self-care may be difficult for caregivers, particularly those who perceive strain.^8^ Although 1 qualitative study^35^ of Black women with diabetes reported that caregiving responsibilities were viewed as a barrier to participants’ diabetes self-care,^35^ quantitative data are limited.

To address this gap and to gain a better understanding of the association of caregiving with caregivers’ health, we examined the association between perceived caregiver strain and diabetes self-care among unpaid caregivers with diabetes, hypothesizing that those with higher levels of caregiver strain would report worse self-care. In addition, we assessed whether the association of caregiving strain with self-care varied by caregivers’ race and sex because existing literature^15,35,36,37^ has reported differences in the association by these demographic characteristics.

## Methods

### Study Cohort

For this cohort study, a cross-sectional analysis was conducted between July 13, 2018, and June 25, 2020, using data from the Reasons for Geographic and Racial Differences in Stroke (REGARDS) study, an ongoing, prospective cohort study evaluating racial and geographic disparities in stroke mortality. Details of the REGARDS study have been described previously.^38^ Recruitment for that study occurred between January 2003 and October 2007 by mail and telephone and was followed by in-home examination. Black individuals and residents of the Stroke Belt, an area in the southeastern US with a high stroke mortality rate, were oversampled by design.^38^ Participants completed a telephone interview in which their medical history was ascertained. Race was defined by self-report as either Black or White. Those reporting other race/ethnic membership were excluded from the REGARDS study.^38^ In-home examination included assessment of blood pressure, height and weight, anthropomorphic measures, and medications. Blood and urine samples were obtained, and electrocardiography was performed for each participant. At 6-month intervals, participants were contacted by phone and asked about hospitalizations and general health status. For the present study, only baseline data (including findings from initial in-home examinations) were used. This study was approved by the institutional review boards of participating institutions, including the University of Alabama Birmingham and Weill Cornell Medicine. All participants provided written informed consent. This study followed the Strengthening the Reporting of Observational Studies in Epidemiology (STROBE) reporting guideline.^39^

The present study included REGARDS study participants aged 45 years or older who reported having diabetes and answered “yes” to the question “Are you currently providing care on an ongoing basis to a family member with a chronic illness or disability? This would include any kind of help such as watching your family member, dressing or bathing this person, arranging care, or providing transportation.”^26^ Diabetes status was ascertained at baseline, defined as an affirmative response to the question, “Has a doctor or other health professional ever told you that you had diabetes or high blood sugar?”^40,41^

### Main Exposure

Caregiver strain was assessed with the question “How much of a mental or emotional strain is it to provide this care?” Responses were categorized as no strain, some strain, or high strain. In prior studies, this measure has been shown to be effective in evaluating the levels of mental and emotional strain associated with caregiving.^15,19,20,37^

### Main Outcomes

Diabetes self-care was assessed across 4 domains derived from the literature.^42,43^ These were Mediterranean diet, physical activity, smoking, and medications.

#### Mediterranean Diet

Evaluation of diet was ascertained at baseline with the Food Frequency Questionnaire, a validated method for evaluating dietary intake.^44^ Responses range from 0 to 9. A Mediterranean diet score was created based on the Food Frequency Questionnaire data. Scores were categorized as low adherence (0-5, suboptimal) vs high adherence (6-9, optimal), as in prior studies.^45^

#### Physical Activity

Physical activity was assessed with the question “How many times per week do you engage in intense physical activity, enough to work up a sweat?” Responses were categorized as 0 to 3 times per week (suboptimal) or greater than or equal to 4 times per week (optimal).^46,47^

#### Smoking Status

Current smoking was defined as a response of “yes” to both of the following questions: “Have you smoked at least 100 cigarettes in your lifetime?” and “Do you smoke cigarettes now, even occasionally?” Nonsmoking was defined by a response of “no” to either question.

#### Medication Adherence

Self-reported medication adherence was assessed using a validated 4-item scale,^48^ with each “yes” response counted, leading to a total adherence score that ranges from 0 to 4. Responses were categorized as low adherence (1-4, suboptimal) and high adherence (0, optimal).^49^

#### Overall Self-Care

We created a composite self-care score, which summed performance across these 4 individual domains, with a score of 0 indicating suboptimal and a score of 1 indicating optimal self-care behavior. Thus, total scores ranged from 0 to 4. Scores were categorized as low (0-2) and high (3-4) overall self-care.

### Covariates

Demographic data were collected, including self-reported age (continuous, in years), sex (male vs female), race (Black vs White), education (less than high school vs high school diploma), annual household income, and region of residence. Regions of residence were designated as the Stroke Buckle (the coastal regions of North and South Carolina and Georgia), the Stroke Belt (the remainder of North and South Carolina and Georgia plus Alabama, Mississippi, Louisiana, Arkansas, and Tennessee), or outside the Stroke Belt (the remaining contiguous 40 states in the US).

Baseline clinical data included a self-reported history of coronary heart disease or electrocardiographic evidence of previous myocardial infarction, self-reported hypertension or systolic blood pressure greater than or equal to 140 mm Hg or diastolic blood pressure greater than or equal to 90 mm Hg, dyslipidemia (defined as total cholesterol level ≥240 mg/dL, low-density lipoprotein level ≥160 mg/dL, or current use of cholesterol-lowering medications), and obesity, defined as a body mass index (calculated as weight in kilograms divided by height in meters squared) greater than or equal to 30. Symptoms of depression were assessed using the 4-item version of the Center for Epidemiological Studies Depression Scale.^36^ This scale asks individuals to rate the number of days over the past week on which they (1) felt depressed, (2) felt lonely, (3) had crying spells, and (4) felt sad. Response options include less than 1 day, 1 to 2 days, 3 to 4 days, and 5 to 7 days (with values of 0, 1, 2, and 3 points, respectively). Elevated symptoms of depression were defined as a score of greater than or equal to 4.^50^ Self-reported physical and mental health were assessed using the Physical and Mental Composite Health Scores from the 12-item Short-Form Health Survey.^51^ The 4-item Perceived Stress Scale was used to determine the level of perceived stress. The 4-item Perceived Stress Scale is a validated measure of participants’ perceptions that their life is unpredictable, uncontrollable, and/or overcommitted. Scores range from 0 to 16. Elevated stress was defined as a score on the 4-item Perceived Stress Scale of 8 or greater.^52^ Social support was assessed using self-reported marital status and the presence or absence of a social network, defined as a report of having seen either no friends or relatives or only 1 friend or relative in the past month.^53^ Additional caregiver characteristics were also collected; participants who responded affirmatively to being caregivers were asked about their social support system and (1) whether they lived with the care recipient, (2) how this person was related to them (eg, spouse, parent), and (3) how many hours per week they spent providing care to this person (<10, 10-19, 20-29, or >30 hours).

### Statistical Analysis

Demographic and clinical characteristics of participants were compared by level of caregiver strain using a χ^2^ test, analysis of variance, and the Kruskal-Wallis test. The associations between caregiver strain and diabetes self-care domains were assessed using the Mantel-Haenszel χ^2^ test for trend. The associations between individual domains and the diabetes self-care score were further examined using multivariable Poisson regression, adjusting for demographic, clinical, physical and mental health, and caregiving covariates. All main outcomes were dichotomous, and some were common (>10% of sample). As such, robust Poisson regression was used to allow for the reporting of a reliable prevalence ratio (PR) for a dichotomous outcome.^54^ Covariates were added to the model in a stepwise fashion, and PRs and 95% CIs were calculated. Effect modification on the association between caregiver strain and diabetes self-care was examined by race and sex. A Wald test was used to evaluate the significance of the interactions. A multiple imputation with chained equations was used to handle missing data. Thirty imputations of the data set were created, and the estimates from individual imputations were combined using Rubin rules.^55^ Variables with the greatest amount of missing data included income (12.0%) and hours spent caregiving (10%). All other covariates had less than 5% of data missing. All analyses were performed using SAS, version 9.4 (SAS Institute) and Stata, version 14 (StataCorp LLC). Crude and fully adjusted associations were calculated using 2-sided Poisson and logistic regression. *P* < .05 was considered statistically significant.

## Results

The main analytic cohort comprised 795 participants with diabetes (Figure). Participants’ demographic and clinical characteristics are presented in Table 1. The mean (SD) age of the cohort was 63.7 (8.6) years; 469 (59.0%) were women, and 452 (56.9%) were Black. Most of the participants had a high school education or greater. With respect to medical comorbidities, 617 (77.7%) had hypertension, 553 (72.1%) had dyslipidemia, 492 (62.3%) were obese, and 186 (23.8%) had a history of coronary heart disease. A total of 605 participants (99.2%) were taking some form of glucose-lowering medication; 514 (84.3%) reported oral medication use, and 172 (28.2%) reported insulin use.

**Figure.**
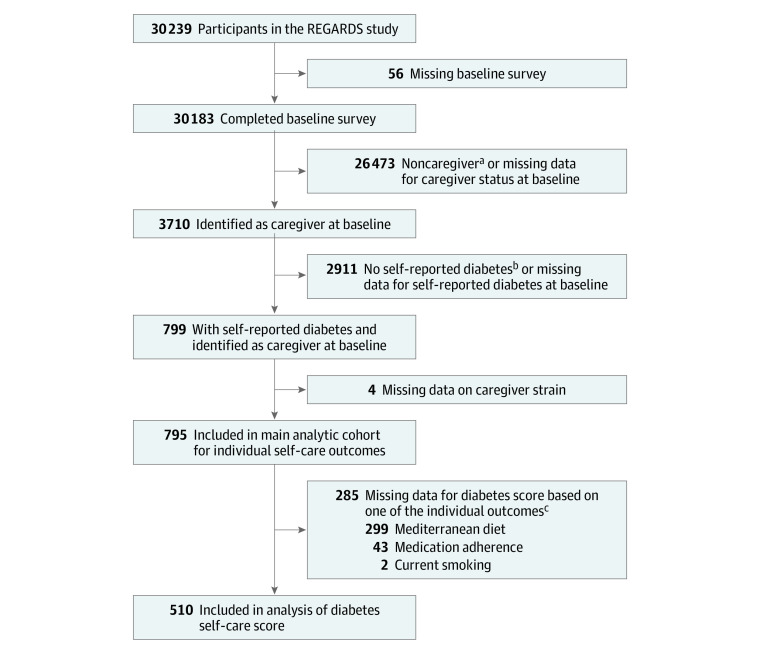
Flow Diagram of the Study Cohort ^a^Caregiver was defined as a participant who answered “yes” to “Are you currently providing care on an ongoing basis to a family member with a chronic illness or disability? This would include any kind of help such as watching your family member, dressing or bathing this person, arranging care, or providing transportation.” ^b^A person with self-reported diabetes was defined as a participant who answered “yes” to “Has a doctor or other health professional ever told you that you had diabetes or high blood sugar?” ^c^Categories are not mutually exclusive. Participants with missing data for an outcome that was included in the diabetes score were placed in the high strain (score ≥3) category if they had a score of at least 3 despite a missing response.

**Table 1.** Characteristics of Caregivers With Diabetes by Level of Caregiver Strain in the REGARDS Cohort

Characteristic	Caregivers^a^				*P* value
	Total (N = 795)	No strain (n = 284)	Some strain (n = 365)	High strain (n = 146)	
Sociodemographic					
Age, mean (SD), y	63.7 (8.6)	64.5 (8.7)	62.9 (8.3)	64.2 (8.9)	.045
Women	469 (59.0)	126 (44.4)	231 (63.3)	112 (76.7)	<.001
Black	452 (56.9)	171 (60.2)	202 (55.3)	79 (54.1)	.35
Low educational attainment (less than high school diploma)	122 (15.4)	52 (18.4)	49 (13.4)	21 (14.4)	.21
Income					
≥$35 000 annually	301 (37.9)	114 (40.1)	144 (39.5)	43 (29.5)	.03
<$35 000 annually	397 (49.9)	128 (45.1)	180 (49.3)	89 (61.0)	
Declined to report	97 (12.2)	42 (14.8)	41 (11.2)	14 (9.6)	
Region of residence					
Non–Stroke Belt	313 (39.4)	119 (41.9)	139 (38.1)	55 (37.7)	.85
Stroke Belt	313 (39.4)	109 (38.4)	145 (39.7)	59 (40.4)	
Stroke Buckle	169 (21.3)	56 (19.7)	81 (22.2)	32 (21.9)	
History of medical conditions					
Coronary heart disease	186 (23.8)	64 (23.0)	84 (23.3)	38 (26.8)	.66
Obese	492 (62.3)	174 (61.5)	225 (62.2)	93 (64.1)	.86
Hypertension	617 (77.7)	220 (77.5)	280 (76.7)	117 (80.7)	.62
Dyslipidemia	553 (72.1)	196 (71.0)	261 (74.4)	96 (68.6)	.38
Medication use					
Oral antiglycemic and/or insulin use	605 (99.2)	228 (98.7)	276 (99.3)	101 (100.0)	.47
Insulin use	172 (28.2)	62 (26.8)	78 (28.1)	32 (31.7)	.66
Oral antiglycemic medication use	514 (84.3)	193 (83.5)	239 (86.0)	82 (81.2)	.49
Antihypertensive use	570 (74.3)	201 (73.6)	260 (73.9)	109 (76.8)	.76
Statin use	359 (45.2)	130 (45.8)	171 (47.0)	58 (39.7)	.32
Physical and mental health					
MCS-Mental Health, median (IQR)	55.1 (47.7-58.6)	57.4 (52.9-60.0)	54.7 (47.7-58.6)	47.0 (37.4-54.5)	<.001
PCS-Physical Health, median (IQR)	44.1 (34.3-52.0)	46.1 (37.5-52.8)	43.9 (34.3-52.0)	39.7 (28.7-50.8)	.003
Depressive symptoms	129 (16.3)	25 (8.9)	51 (14.1)	53 (36.3)	<.001
Perceived stress, median (IQR)	3.0 (1.0-6.0)	2.0 (0.0-4.0)	4.0 (1.0-6.0)	7.0 (4.0-8.0)	<.001
Social support					
Not married	266 (33.5)	86 (30.3)	124 (34.0)	56 (38.4)	.23
No social network	105 (13.4)	32 (11.4)	43 (11.9)	30 (20.8)	.001
Characteristics of caregiving					
Living with care recipient	454 (57.1)	151 (53.2)	212 (58.1)	91 (62.3)	.17
Relationship to caregiver					
Parent	210 (26.4)	68 (23.9)	111 (30.4)	31 (21.4)	.047
Spouse	200 (25.2)	66 (23.2)	85 (23.3)	49 (33.8)	
Child	117 (14.7)	42 (14.8)	57 (15.6)	18 (12.4)	
Sibling	80 (10.1)	30 (10.6)	31 (8.5)	19 (13.1)	
Other	187 (23.6)	78 (27.5)	81 (22.2)	28 (19.3)	
Caregiving hours per wk					
<10	263 (37.5)	128 (51.4)	111 (34.4)	24 (18.5)	<.001
10-19	117 (16.7)	40 (16.1)	56 (17.3)	21 (16.2)	
20-29	86 (12.3)	27 (10.8)	44 (13.6)	15 (11.5)	
≥30	236 (33.6)	54 (21.7)	112 (34.7)	70 (53.8)	

Abbreviations: IQR, interquartile range; MCS, Mental Composite Summary Score; PCS, Physical Composite Summary Score; REGARDS, Reasons for Geographic and Racial Differences in Stroke.

^a^ Data are presented as number (percentage) of caregivers unless otherwise indicated.

A total of 146 participants (18.4%) reported a high level of caregiver strain, 365 (45.9%) reported some strain, and 284 (35.7%) reported no strain. Participants with a high level of caregiver strain were more likely to be women, have low income, have high levels of perceived stress, lack social support, report worse mental and physical health, have more depressive symptoms, and spend 30 or more hours per week caregiving.

Compared with participants who reported no strain or some strain, those who reported a high level of caregiver strain were less likely to exercise 4 or more times per week (28 [19.2%] with high strain vs 82 [28.9%] with no strain vs 96 [26.3%] with some strain) and have high adherence to their medication regimen (80 [57.1%] with high strain vs 192 [71.4%] with no strain vs 211 [61.5%] with some strain). In addition, compared with participants who reported no strain or some strain, those who reported a high level of caregiver strain were more likely to have low overall self-care scores (71 [75.5%] with high strain vs 112 [62.6%] with no strain vs 155 [65.4%] with some strain) (Table 2). Although participants with a high level of strain were less likely to report high adherence to the Mediterranean diet, the differences were not statistically significant. No difference in smoking status was observed between caregiver strain groups.

**Table 2.** Diabetes Self-Care Domains by Level of Caregiver Strain

Characteristic	Points	Caregivers, No (%)				*P* value^a^
		Total (N = 795)	No strain (n = 284)	Some strain (n = 365)	High strain (n = 146)	
**Health behavior**						
Exercise, times per wk						
0-3	0	589 (74.1)	202 (71.1)	269 (73.7)	118 (80.8)	.04
≥4	1	206 (25.9)	82 (28.9)	96 (26.3)	28 (19.2)	
Mediterranean diet adherence						
Low, 0-5	0	390 (78.6)	141 (83.9)	175 (74.5)	74 (79.6)	.22
High, 6-9	1	106 (21.4)	27 (16.1)	60 (25.5)	19 (20.4)	
Medication adherence						
Low, 1-4	0	269 (35.8)	77 (28.6)	132 (38.5)	60 (42.9)	.006
High, 0	1	483 (64.2)	192 (71.4)	211 (61.5)	80 (57.1)	
Smoking status						
Current smoker	0	102 (12.9)	37 (13.1)	45 (12.4)	20 (13.7)	.93
Not current smoker	1	691 (87.1)	246 (86.9)	319 (87.6)	126 (86.3)	
**Diabetes self-care score**^b^						
Self-care score						
Low, <3	NA	338 (66.3)	112 (62.6)	155 (65.4)	71 (75.5)	.04
High, ≥3	NA	172 (33.7)	67 (37.4)	82 (34.6)	23 (24.5)	

Abbreviation: NA, not applicable.

^a^ Mantel-Haenszel χ^2^ test for trend.

^b^ A total of 510 participants were included in the calculation of a composite diabetes self-care score.

Unadjusted models revealed consistent findings (Table 3). However, after adjusting for sociodemographic factors; medical conditions; and overall physical and mental health, social support, and caregiving characteristics, the association between a high level of caregiver strain and less physical activity was attenuated. In the fully adjusted model, strain (some and high) remained significantly associated with less medication adherence (adjusted PR [aPR]: some strain, 0.88 [95% CI, 0.78-0.99]; high strain, 0.83 [95% CI, 0.69-0.99]) (Table 3). Of note, some caregiver strain was associated with high adherence to a Mediterranean diet in both unadjusted (PR, 1.59; 95% CI, 1.06-2.39) and adjusted models (aPR, 1.90; 95% CI, 1.24-2.92). There was no association of a high level of strain with adherence to the Mediterranean diet in the adjusted model (aPR, 1.75; 95% CI, 0.98-3.11). The association between a high level of caregiver strain and lower overall self-care was attenuated after adjustment for sociodemographic data (Table 4).

**Table 3.** Association Between Caregiver Strain and Diabetes Self-Care Health Behaviors Among REGARDS Participants With Diabetes Who Were Caregivers

Caregiver strain	Prevalence ratio (95% CI)						
	Unadjusted	Model 1^a^	Model 2^b^	Model 3^c^	Model 4^d^	Model 5^e^	Model 6^f^
High physical activity							
No strain	1 [Reference]	1 [Reference]	1 [Reference]	1 [Reference]	1 [Reference]	1 [Reference]	1 [Reference]
Some strain	0.91 (0.71-1.17)	0.95 (0.73-1.22)	0.95 (0.74-1.22)	0.95 (0.74-1.23)	0.97 (0.76-1.25)	0.95 (0.74-1.22)	0.95 (0.73-1.22)
High strain	0.66 (0.45-0.97)	0.70 (0.47-1.03)	0.73 (0.50-1.06)	0.75 (0.51-1.09)	0.77 (0.53-1.13)	0.72 (0.49-1.06)	0.71 (0.47-1.08)
High Mediterranean diet adherence							
No strain	1 [Reference]	1 [Reference]	1 [Reference]	1 [Reference]	1 [Reference]	1 [Reference]	1 [Reference]
Some strain	1.59 (1.06-2.39)	1.70 (1.13-2.58)	1.80 (1.19-2.71)	1.80 (1.19-2.71)	1.87 (1.25-2.81)	1.94 (1.27-2.97)	1.90 (1.24-2.92)
High strain	1.27 (0.75-2.16)	1.38 (0.81-2.36)	1.48 (0.88-2.50)	1.50 (0.88-2.54)	1.64 (0.97-2.78)	1.81 (1.04-3.15)	1.75 (0.98-3.11)
Not currently smoking							
No strain	1 [Reference]	1 [Reference]	1 [Reference]	1 [Reference]	1 [Reference]	1 [Reference]	1 [Reference]
Some strain	1.01 (0.95-1.07)	1.01 (0.95-1.07)	1.01 (0.95-1.07)	1.01 (0.95-1.07)	1.01 (0.95-1.07)	1.01 (0.95-1.08)	1.02 (0.96-1.08)
High strain	0.99 (0.92-1.07)	0.98 (0.90-1.06)	0.98 (0.91-1.07)	0.99 (0.91-1.07)	0.99 (0.91-1.07)	0.99 (0.91-1.07)	1.02 (0.93-1.11)
High medication adherence							
No strain	1 [Reference]	1 [Reference]	1 [Reference]	1 [Reference]	1 [Reference]	1 [Reference]	1 [Reference]
Some strain	0.86 (0.77-0.96)	0.86 (0.76-0.96)	0.87 (0.77-0.97)	0.86 (0.77-0.96)	0.86 (0.77-0.97)	0.88 (0.78-0.99)	0.88 (0.78-0.99)
High strain	0.80 (0.68-0.94)	0.79 (0.67-0.93)	0.81 (0.68-0.95)	0.81 (0.68-0.95)	0.81 (0.68-0.95)	0.83 (0.70-0.99)	0.83 (0.69-0.99)

Abbreviation: REGARDS, Reasons for Geographic And Racial Differences in Stroke.

^a^ Model 1 was adjusted for sociodemographic data (age, sex, race, education, income, and geographic region).

^b^ Model 2 adjusted for the variables in model 1 plus medical conditions (history of heart disease, hypertension, obesity, and dyslipidemia) and physical health.

^c^ Model 3 adjusted for the variables in model 2 plus social support (marital status, social network).

^d^ Model 4 adjusted for the variables in model 3 plus caregiver strain data (living with care recipient, relationship to care recipient).

^e^ Model 5 adjusted for variables in model 4 plus hours spent caregiving.

^f^ Model 6 adjusted for variables in model 5 plus mental health (12-item Short Form Health Survey, Mental Composite Summary Score), depressive symptoms (the Center for Epidemiological Studies-Depression scale), and perceived stress (4-item Perceived Stress Scale).

**Table 4.** Association Between Caregiver Strain and Composite Diabetes Self-Care Score Among REGARDS Participants With Diabetes Who Were Caregivers^a^

Caregiver strain	Prevalence ratio (95% CI)						
	Unadjusted	Model 1^b^	Model 2^c^	Model 3^d^	Model 4^e^	Model 5^f^	Model 6^g^
No strain	1 [Reference]	1 [Reference]	1 [Reference]	1 [Reference]	1 [Reference]	1 [Reference]	1 [Reference]
Some strain	0.92 (0.71-1.20)	1.02 (0.78-1.33)	1.07 (0.82-1.38)	1.07 (0.83-1.39)	1.08 (0.84-1.40)	1.05 (0.80-1.37)	1.05 (0.80-1.37)
High strain	0.65 (0.44-0.98)	0.73 (0.48-1.10)	0.79 (0.53-1.17)	0.81 (0.55-1.21)	0.87 (0.58-1.29)	0.81 (0.53-1.22)	0.82 (0.53-1.27)

Abbreviation: REGARDS, Reasons for Geographic and Racial Differences in Stroke.

^a^ A sample size of 510 was used only for the composite diabetes self-care score outcome (1 of the 4 outcomes in this study); this was less than 795 (the main sample size) owing to missing data, primarily from the Mediterranean diet (1 of the components of the score). To preserve power for the individual self-care outcomes, a sample size of 795 was used.

^b^ Model 1 was adjusted for sociodemographic data (age, sex, race, education, income, and geographic region).

^c^ Model 2 adjusted for the variables in model 1 plus medical conditions (history of heart disease, hypertension, obesity, and dyslipidemia) and physical health.

^d^ Model 3 adjusted for the variables in model 2 plus social support (marital status, social network).

^e^ Model 4 adjusted for the variables in model 3 plus caregiver strain data (living with care recipient, relationship to care recipient).

^f^ Model 5 adjusted for variables in model 4 plus hours spent caregiving.

^g^ Model 6 adjusted for variables in model 5 plus mental health (12-item Short Form Health Survey, Mental Composite Summary Score), depressive symptoms (the Center for Epidemiological Studies-Depression scale), and perceived stress (4-item Perceived Stress Scale).

We tested for interactions by race and sex with the association between caregiver strain and diabetes self-care domains (eTable 1 in the Supplement). No interactions were significant except for race and smoking status. Among White individuals, some caregiving strain was crudely associated with not smoking. In contrast, among Black individuals, there was no association between caregiver strain and smoking. However, in the final model, the association between some strain and caregiving among White individuals was attenuated (eTable 2 in the Supplement).

## Discussion

In this nationally representative cross-sectional study of US caregivers with diabetes, we found that 18.4% of participants reported a high level of caregiver strain. A high level of caregiver strain was associated with lower levels of physical activity and medication adherence as well as worse self-care overall compared with no strain or some caregiver strain. In adjusted analyses, the association between a high level of caregiver strain and low medication adherence remained statistically significant. In addition, some caregiver strain was associated with greater likelihood of high adherence to the Mediterranean diet compared with no strain or a high level of strain. Although we expected to find differences in these associations by race and sex, few such moderated differences were observed. This is notable because in prior studies, different subgroups were found to have greater risk of poor health outcomes associated with strain.^15,16,35^ Future studies should address sex and racial differences in reports of strain and health-related behaviors to gain a better understanding of these variable findings.

Our findings contribute to the current body of literature, which has revealed that caregiving may be associated with worse health outcomes in certain subsets of caregivers.^4,7,8,9,10,11,12,13,14,15,16,17^ The rationale is that caregivers with higher levels of strain have less time to maintain their own health and well-being. We applied these findings to diabetes, a highly prevalent disease that requires a high degree of self-care. Several plausible reasons for our findings deserve additional investigation. First, the more strain that caregivers experience, the less time they have for themselves. For example, a study of healthy middle-aged caregivers^56^ revealed that those with greater caregiving demands (eg, more individuals to care for, more hours spent caregiving per week) had less time for their own personal health needs. Second, strained caregivers may also assign more importance to the health of others than their own.^56^ In our study, caregivers reporting some strain had high adherence to the Mediterranean diet. Results of previous studies of nutrition in families and spousal pairs have suggested that health behavior interventions for 1 spouse can positively influence behavior of the other, particularly with regard to changes in family diet.^57,58,59^ In studies of spousal pairs in which 1 member had diabetes, the collective effort of both partners was found to be associated with better dietary adherence.^60^ This set of caregivers may have experienced a ripple effect, that is, their preparation of healthy meals for their care recipients may have led to a healthier food environment for themselves. Future longitudinal studies should seek to test these hypotheses and elucidate the underlying mechanisms that can explain the association between caregiver strain and self-care among caregivers with diabetes.

In adjusted analyses, we found that for some outcomes (physical activity, Mediterranean diet, and smoking status), the association with strain was attenuated after adjusting for sociodemographic factors; medical conditions; and overall physical and mental health, social support, and caregiving characteristics. Of note, however, for medication adherence, the association between a high level of strain and poor adherence persisted after these adjustments were made. Medication adherence is a critical component of diabetes self-care. Difficulty with medication adherence is often multifactorial because cost of medications, access to care, health beliefs regarding the safety and effectiveness of medications, and health literacy and numeracy are associated with adherence.^61^ However, beyond these underlying determinants, caregivers experiencing a high level of strain with varying responsibilities may not prioritize medication adherence, especially if medications need to be taken at certain times of the day or if a regimen is complex. In addition, although we were unable to assess health care utilization, caregivers experiencing a high level of strain may be less likely to prioritize attending their own physician appointments, and thus, opportunities to reinforce the need for medication adherence may be lost. Similarly, caregivers may not prioritize their own physical activity over their caregiving demands. Screening for caregiver strain among patients with diabetes warrants consideration by health care professionals because this may provide information on a patient’s ability to perform his or her own self-care tasks.

Our study has implications for the care of both patients and caregivers. We found that the prevalence of caregiving strain was high, with 45.9% of caregivers reporting some strain and 18.4% reporting a high level of strain. The subset of caregivers experiencing a high level of strain appeared to be at higher risk for poor health outcomes, as reported in previous studies.^17,18^ Millions of caregivers in the US are already providing unpaid care, and as the population ages, the need for unpaid caregiving will continue to increase.^62^ Moreover, as usual routes to care have been altered or put on hold during the COVID-19 pandemic, caregivers have continued to provide care, often in relative isolation and at times with risks to their own health, which may be associated with increased levels of caregiver strain.

At present, medical encounters in primary care are structured to prioritize patients and their needs. Although appropriate, this model often fails to engage and integrate caregivers who may be playing a vital role for patients in the home in addition to contending with their own medical conditions, which may impact their own and the care recipients’ health. Furthermore, the degree to which caregivers are involved or strained while providing care often goes unassessed.^4,63^ Increased awareness of caregiver strain among caregivers with diabetes appears to be needed by medical professionals because a high level of strain may be associated with alterations in caregivers’ behavior related to their own health. In addition to broad screening for high levels of strain, future interventions might also seek to provide additional support to caregivers with diabetes.

### Strengths and Limitations

This study has strengths. The study included a large, geographically diverse cohort and oversampling of Black participants and had the ability to assess caregiver strain and characteristics among caregivers with diabetes. Furthermore, we were able to assess several domains of diabetes self-care.

This study also has limitations. Data collection occurred at 1 point in time. Therefore, we were unable to account for changes in caregiving intensity or strain or for changes in self-care behaviors that can occur for an individual throughout life. Second, diabetes status was ascertained at baseline by self-report of a physician diagnosis. Third, a small sample size of 510 used in the diabetes self-care score limits the power of this analysis.

## Conclusions

In this cohort study of US adult caregivers with diabetes, a high level of caregiver strain was associated with worse self-care, particularly with respect to medication adherence, suggesting that high caregiving burden may interfere with caregivers’ self-care behaviors. Given the prevalence of caregiving in the US, increased awareness of the association of caregiver strain with self-care appears to be needed, particularly among those who have diabetes. Additional, prospective studies are needed to examine the association of our findings with health outcomes among caregivers with diabetes.

## Notes

Supplement. **eTable 1.** Association Between Caregiver Strain and Diabetes Self-Care Domains: Interactions by Race and Gender **eTable 2.** Effect of Caregiver Strain on Smoking Status among 793 REGARDS Participants, Stratified by Race
