# Supplementary material for: Association Between Caregiver Strain and Self-Care Among Caregivers With Diabetes
Source: JAMA Netw Open. 2021 Feb 11;4(2):e2036676. doi: 10.1001/jamanetworkopen.2020.36676 (PMC7879235; doi:10.1001/jamanetworkopen.2020.36676)
Supplement: Supplement. — eTable 1. Association Between Caregiver Strain and Diabetes Self-Care Domains: Interactions by Race and Gender eTable 2. Effect of Caregiver Strain on Smoking Status among 793 REGARDS Participants, Stratified by Race [file jamanetwopen-e2036676-s001.pdf]

Supplementary Online Content

King A, Bryan Ringel JB, Safford MM, et al. Association between caregiver strain and self-care among caregivers with diabetes. *JAMA Netw Open*. 2021;4(2): e2036676. doi: 10.1001/jamanetworkopen.2020.36676

eTable 1. Association Between Caregiver Strain and Diabetes Self-Care Domains: Interactions by Race and Gender

eTable 2. Effect of Caregiver Strain on Smoking Status among 793 REGARDS Participants, Stratified by Race

This supplementary material has been provided by the authors to give readers additional information about their work.

© 2021 King A et al. *JAMA Network Open*.

eTable 1. Association Between Caregiver Strain and Diabetes Self-Care Domains: Interactions by Race and Gender

Self-Care Behaviors	Gender		Race	
	Unadjusted P for interaction	Full Model* P for interaction	Unadjusted P for interaction	Full Model* P for interaction
Physical Activity	0.56	0.46	0.96	0.99
Mediterranean diet	0.20	0.24	0.51	0.52
Smoking	0.17	0.14	**0.02***	**0.01***
Medication adherence	0.79	0.90	0.23	0.36
Diabetes self-care score	0.50	0.32	0.60	0.49

*Fully Adjusted Model: Sociodemographic Data + Medical Conditions + Social Support + Caregiver Strain Data + Hours spent caregiving + Mental health (SF-12, MCS), Depressive symptoms (CES-D4) and Perceived Stress (PSS)

eTable 2. Effect of Caregiver Strain on Smoking Status among 793 REGARDS Participants, Stratified by Race

Caregiver strain	Unadjusted	Fully Adjusted*
	PR (95% CI)	PR (95% CI)
White (n = 343)		
Not Currently smoking		
No strain	REF	REF
Some strain	0.92 [0.85,0.99]	0.94 [0.87,1.02]
High Strain	0.92 [0.83,1.03]	0.94 [0.83,1.08]
Black (n = 450)		
Not Currently smoking		
No strain	REF	REF
Some strain	1.08 [0.99,1.18]	1.08 [0.98,1.18]
High Strain	1.05 [0.93,1.17]	1.06 [0.94,1.20]

Abbreviations: PR - Prevalence Ratio; CI- Confidence Interval

*Fully Adjusted Model: Sociodemographic Data + Medical Conditions + Social Support + Caregiver Strain Data + Hours Spent caregiving + Mental health (SF-12, MCS), Depressive symptoms (CES-D4) and Perceived Stress (PSS)

© 2021 King A et al. *JAMA Network Open*.
